# Recent emergence of vaccine-derived poliovirus type 2 in Senegal and virus spread to neighboring countries

**DOI:** 10.1016/j.ijid.2025.108122

**Published:** 2025-12

**Authors:** Ndack Ndiaye, Boly Diop, Ousmane Kébé, Serigne Fallou MBacké NGom, Landy Gerald Boussiengui, Annick Dosseh, Abdoulaye Sam, Mohamed Dia, Baba Sy, Aliou Diallo, Thierno Ndiaga Gueye, Papa Chimére Diaw, Ibrahima Oumar Ba, Julius E. Chia, Jean Pierre Diallo, Boubacar Diallo, Yoro Sall, Amadou Alpha Sall, Ousmane Faye, Ndongo Dia, Ousmane Madiagne Diop, Abdourahmane Sow, Kfutwah Anfumbom, Martin Faye

**Affiliations:** 1 Virology Department, Institut Pasteur de Dakar, 220 Dakar, Senegal; 2 Prevention office, Ministry of Health and Social actions, 4024 Dakar, Senegal; 3 World Health Organization, Inter-Country Support Team for Western Africa, 7019 Ouagadougou, Burkina Faso; 4 World Health Organization, Senegal Country Office, 4039 Dakar, Senegal; 5 World Health Organization, AFRO, 06 Brazzaville, Republic of Congo; 6 Public health department, Institut Pasteur de Dakar, 220 Dakar, Senegal; 7 The Polio Eradication Department, World Health Organization, 1211 Geneva, Switzerland

**Keywords:** Acute flaccid paralysis, Environmental surveillance, cVDPV2, NIE-ZAS-1 emergence group, Senegal

## Abstract

**Objectives:**

As we approach the eradication of poliomyelitis, one of the global challenges is to control and interrupt the transmission of the vaccine-derived polioviruses. In Senegal, an outbreak of circulating vaccine-derived poliovirus type 2 (cVDPV2) of the NIE-JIS-1 emergence group was previously reported from 2020 to 2021.

**Methods:**

Our study report a newemergence of cVDPV2 in Senegal from 2023 to 2024, first detected in sewage and later in stool samples from children with acute flaccid paralysis, particularly, in Southeastern Senegal.

**Results:**

All recent strains from Senegal were genetically linked, and one sequence was linked to the type 2 novel oral polio vaccine. The phylodynamic analysis showed that the 2023-2024 cVDPV2 sequences from Senegal grouped into a monophyletic group with strains from the Republic of Guinea, and belonged to the NIE-ZAS-1 emergence group. Our data also show that the NIE-ZAS-1 virus was introduced to Senegal around 2010 (95% Highest Posterior Density, 2003-2017) and recently spread to the Republic of Guinea and the Republic of Mauritania in 2023 while it continuously circulated in Senegal between 2023 and 2024.

**Conclusion:**

Our data highlight the importance of strengthening the poliovirus vaccine coverage and surveillance in these countries characterized by frequent population movements. Moreover, more studies on risks associated with VDPV2 mutations to enhance virulence are warranted.

## Highlights

• Re-emergence of circulating vaccine-derived poliovirus type 2 belonging to the NIE-ZAS-1 emergence group in Senegal.
• One sequence linked to the type 2 novel oral polio vaccine.
• Detection of circulating vaccine-derived poliovirus type 2 first diverged in 1974 (95% High Posterior Density range, 1938-2010).
• Importance needs of strengthening poliovirus vaccine coverage and surveillance.

## Introduction

Currently, one of the major public health concerns worldwide, polioviruses (PVs) consist of three types (PV1, PV2, and PV3), belonging to the enterovirus C species, *Enterovirus* genus, and *Picornaviridae* family [1].

Launched in 1988, the Global Polio Eradication Initiative (GPEI) eradicated two of the three wild polioviruses (WPVs), namely, WPV2 and WPV3, whereas WPV1 remains endemic in Pakistan and Afghanistan [2]. The success of reducing WPV transmission worldwide is due to considerable efforts made through the use of inactivated poliovirus vaccines (IPVs) and the live attenuated oral polio vaccine (OPV). Nevertheless, although the OPV has several advantages, limitations do exist: first, the development of vaccine-associated paralytic poliomyelitis resulting from the mutation and proliferation of polioviruses in vaccinated individuals and those in contact with them; second, the emergence of vaccine-derived polioviruses (VDPVs) related to the divergence from the OPV strains in the viral protein 1 (VP1) region. Indeed, the Sabin vaccine strain can evolve or lose its attenuating mutations during replication in the gut, regaining transmissibility and pathogenicity similar to that of WPVs. VDPVs are classified into three types: circulating (cVDPVs), in evidence of community transmission; immunodeficiency-associated VDPVs, when it is isolated from patients with primary immunodeficiency (PID); and ambiguous, when circulation cannot be confirmed and when the case does not have primary immunodeficiency.

Although VDPVs from OPV types 1 and 3 occur, those from OPV type 2 (VDPV2) are the most common. Since 2005, cVDPV2 outbreaks have been reported in many African and Asian countries [3]. To avoid the inherent risk of cVDPV2 emergence, the OPV type 2 strain was withdrawn globally from trivalent live attenuated OPVs (tOPV) in 2016. Therefore, the detection of VDPV2 is considered as a global public health emergency [3]. Since September 2024, circulating VDPV type 2 (cVDPV2) has been detected in sewage samples within routine and research environmental surveillance (ES) activities in five European countries [4].

In Africa, the GPEI has established a surveillance program for acute flaccid paralysis (AFP) in children aged <15 years and an ES in all countries where applicable for early detection of the circulation of VDPVs and WPVs [3]. Africa was declared free of WPVs in August 2020. However, WPV1 was imported into Malawi and Mozambique in February and May 2022, respectively [2].

In Senegal, stool and sewage samples from AFP cases and the environment, respectively, are routinely tested in the Intercountry Reference Center for Poliomyelitis at the Institut Pasteur de Dakar (IPD) using viral isolation and molecular typing techniques. Between 2020 and 2021, a cVDPV2 outbreak was recorded in the country, linked to the viruses circulating in Guinea [5]. In response to this 2020-2021 outbreak in Senegal, a localized vaccination campaign using the IPV was carried out, followed by two mass vaccination campaigns using the type 2 novel OPV (nOPV2). These vaccination activities played a significant role in interrupting the virus transmission. The last cVDPV2 confirmed from a contact was identified on November 19, 2021. To strengthen the surveillance and monitoring of the impact of public health measures, ES was expanded in 2022 from two to 14 collection sites located in seven of the 14 regions in the country. After these measures, no cVDPV2 was detected in Senegal until November 2023. However, there was detection of a cVDPV2 immediately after this period in a sewage sample collected in the Dakar region, indicating probably a new emergence of the virus in the country. Herein, we report on the re-emergence of cVDPV2 in Senegal between 2023 and 2024, through detections from an AFP case and nine ES samples.

## Materials and methods

### Ethical statement

This study was conducted through the GPEI. This study did not involve human participants but rather the use of cell culture isolates of viruses recovered from stool specimens of AFP cases and wastewater samples collected through routine poliomyelitis surveillance activities initiated by the World Health Organization (WHO) for public health purposes. All the technical and ethical aspects were approved by the WHO and the Ministry of Health.

### Sample collection

As recommended by the WHO [6], an identified AFP case must be followed by the collection of two stool samples (S1 and S2) taken 24-48 hours apart within 14 days from the onset of paralysis. Unlike the AFP case surveillance, ES requires 1 L of sewage sample collected once every 2 weeks using defined WHO procedures with strict adherence to safety requirements and transferred into sterile labeled glass bottles.

### Sample processing and poliovirus classification

Primary stool specimens and sewage samples were processed according to the WHO standard procedures [6]. In virus isolation technique, specimens exhibiting complete Cytopathic effect only on L20B cells or L20B and RD were classified as suspected PVs and further subjected to intratypic differentiation assays by reverse transcription–PCR targeting redundant regions in the VP1 region, according to the Centers for Disease Control and Prevention protocol [7].

### Sequencing and genomic data analysis

The isolates with discordant intratypic differentiation results were processed for sequencing using a method based on the Oxford Nanopore technology [8] and analyzed using the Poliovirus Investigation Resource Automating Nanopore Haplotype Analysis [8]. Our data set, including the newly generated VP1 sequences, additional Sabin/VDPVs sequences available at the Intercountry Reference Center for Poliomyelitis at IPD, and previous sequences retrieved from GenBank (https://www.ncbi.nlm.nih.gov/genbank) (accessed on October 12, 2024), were aligned using the “Multiple Alignment using Fast Fourier Transform” (MAFFT) program with default parameters. The occurrence of nucleotide and amino acid polymorphisms on the poliovirus type 2’s VP1 was also assessed using the AY184220 Sabin 2 strain sequence as a reference.

### Phylodynamic reconstructions

Root-to-tip divergence and regression slopes and correlations analysis were performed using the TempEst v1.5.3 [9] software to assess the reliable temporal signal of VDPV2 VP1 sequences. Evolutionary analysis of VDPV2 was also performed using BEAST2 v2.7.6 [10] to estimate the mean substitution rate and the time of the most recent common ancestor (tMRCA). A maximum clade credibility tree was constructed by incorporating a 10% burn-in period. Median heights were generated from a 50-million generation, with a Bayesian Markov chain Monte Carlo approach for phylogenetic reconstruction sampling every 1000 generations. In addition, a general time-reversible nucleotide substitution model with a gamma distribution before each relative substitution rate and a strict clock model were used to infer the VDPV2 evolutionary timescale. The analysis assumed a strict clock model and a coalescent Bayesian Skyline as tree priors. Convergence was confirmed by achieving an effective sample size greater than 200, using Tracer v1.7.2 [11]. The number of viral imports, exports, and persistence between regions was also performed using BEAST2 v2.7.6 [10].

## Results and discussion

VDPVs can cause acute paralysis similar to that associated with WPVs, posing a considerable challenge to public health and the eradication of poliomyelitis. In 2017, large outbreaks attributed to cVDPV2 were reported, primarily, in Africa, followed by Syria, and spanned more than 6 months [12]. This was associated with the waning of natural immunity against poliovirus type 2 as a result of the withdrawal of the type 2 from tOPVs since April 2016. Therefore, individuals remain more susceptible to poliovirus type 2 infections because the IPV used in vaccination campaigns only induces a limited mucosal immunity.

Since that period, the number of outbreaks from cVDPV2 has been increasing worldwide, especially in Africa and the Eastern Mediterranean region where the numbers of zero-dose children are high in some regions. In addition, in 2024, cVDPV2 was detected in environmental samples in Finland, Germany, Poland, Spain, and the United Kingdom [4]; and several detections from AFP cases and environmental samples have also been recently reported in Western countries [12].

**Figure 1:**
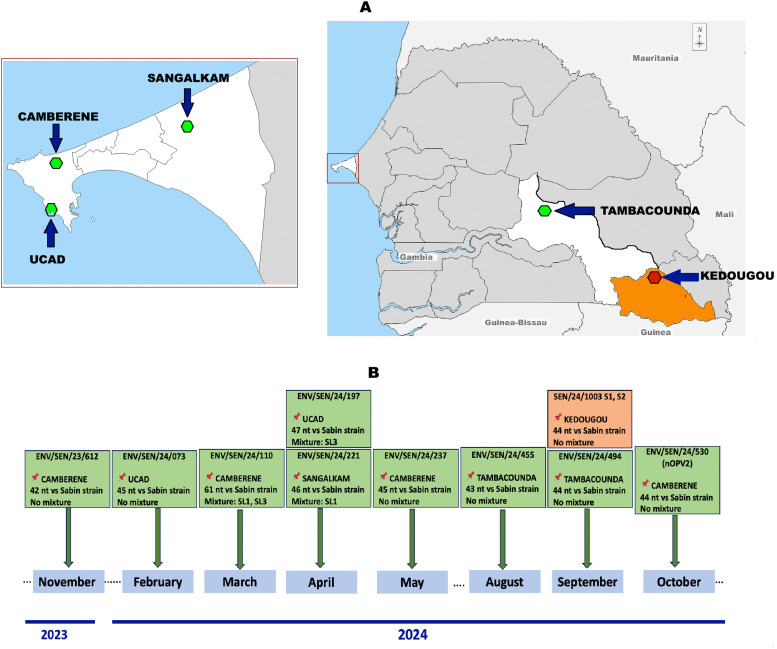
Map showing environmental collection sites (green dot) and the health district (red dot) where circulating vaccine-derived poliovirus type 2 isolates were detected (a) and their characteristics (b) between November 2023 and October 2024.

In Senegal, an emergence of cVDPV2 has been reported from 2020 to 2021 in sewage and AFP cases after 10 years of non-circulation [5]. Afterward, no further detections were reported until November 2023, when a cVDPV2 was found from a sewage sample collected from the Dakar region (Figure 1a, b). Here, we report on the recent re-emergence and outstanding detection of cVDPV2 in Senegal with detections from an AFP case and sewage samples.

From November 2023 to November 2024, a total of 572 stool samples from 287 children with AFP and 229 sewage samples were collected and tested for polioviruses. Overall, 33 suspected polioviruses, including 10 confirmed VDPV2, were identified from four sewage collection sites in three health districts.

The number of VDPV2 isolates increased considerably between February and May 2024, with seven strains isolated from several ES sites in the Dakar region. The detection of VDPV2 in the sewage system could be associated with shedding by asymptomatic individuals, by children who were recently vaccinated with the OPV, or virus importations. Interestingly, VDPV2 isolates were later detected in the Tambacounda and Kédougou regions (Southeastern Senegal), where it was identified from a sewage sample and an AFP case in August and September 2024, respectively (Figure 1a, 1b). In fact, the VDPV2-confirmed case identified in the Kédougou region (SEN-24-1003) was a 1-year-old boy who originated from the Republic of Guinea where an outbreak of cVDPV2 is currently reported. The patient had received no poliovirus vaccine. On September 25, 2024, he was admitted 5 days after the onset of symptoms, including fever and bilateral paralysis of lower limbs. Interestingly, the two VDPV2-positive specimens were collected on September 25-26, 2024 and were closely related to a strain collected in the Republic of Guinea in 2023. Despite a cross-country collaboration for epidemiologic investigations, the patient has been lost to follow-up, suggesting a possible return to the Republic of Guinea, which shares borders with the Kédougou region in Senegal. These data point to the crucial need of strengthening surveillance in the country, urgent initiation of ES in the Kédougou region, and cross-border collaboration between Senegal and the Republic of Guinea through simultaneous vaccination campaigns and cooperation during field investigations in health districts along the borders on both sides.

Although nOPV2 is more genetically stable and less likely to result in VDPV2 than the monovalent oral poliovirus vaccine type 2 (mOPV2)/tOPVs, there is still a risk of reversion [13]. We identified one VDPV2 linked to nOPV2 and VDPV2 in mixtures with Sabin type 1 and/or 3 that were isolated from three sewage samples (Figure 1b), indicating that these viruses were likely secreted by nOPV2/ bivalent oral poliovirus vaccine type (bOPV) vaccine recipients. In early March 2023, seven AFP cases from the Democratic Republic of the Congo and Burundi was associated with cVDPV2 derived from the nOPV2 [13]. Although the OPV vaccine has helped eliminate polioviruses in a part of the world, the problem arises when vaccination coverage is suboptimal. Otherwise, the advantage that OPV can be administered by volunteers does not mitigate the need to use IPV immunization in every country. Despite its parenteral route or higher per-dose vaccine costs, IPVs are not associated with any adverse event after immunization and is completely safe [14].

Currently, almost 30% of all countries in the world have a vaccine coverage of less than 80%, with immunization coverage as low as 36% in some countries [4]. In terms of data completeness and quality, the routine vaccine coverage in Senegal is consistently declining. In fact, since the mass vaccination campaigns in June and December 2021, followed by the last round in February 2022, no supplementary immunization activities (SIAs) have been carried out in the country to date. This observation is particularly alarming, given that gaps in vaccination coverage could contribute to future resurgence of the disease. Thus, additional strategies might be required with high-quality, timely vaccine campaigns after detections or not of new cVDPV2 cases.

Enteroviruses are characterized by a great genetic variability, and Sabin-like polioviruses can genetically evolve into VDPVs (>1% VP1 mutation for type 1 and 3, >0.6% VP1 mutation for type 2). Based on data from the nanopore sequencing at IPD, the newly characterized cVDPV2 sequences showed nucleotide distances ranging from 4.2% to 6.1% in the VP1 gene from the Sabin-like type 2 strains (Figure 1b), probably indicating a sustained VDPV2 circulation. Likewise, the second key determinant of Sabin 2 attenuated phenotype corresponding to an isoleucine-to-threonine substitution (U2909C) at position 145 of the VP1 gene encoding was also identified in all newly characterized cVDPV2 sequences.

However, it is also important to report the occurrence of Sabin-like strains. In fact, early reporting of any Sabin-like strains allows public health authorities to investigate, assess community immunity, and start targeted vaccination campaigns to stop potential appearance and transmission of VDPV. In addition, mutations associated with the nOPV2 were identified, confirming its origin as a vaccine strain.

**Figure 2:**
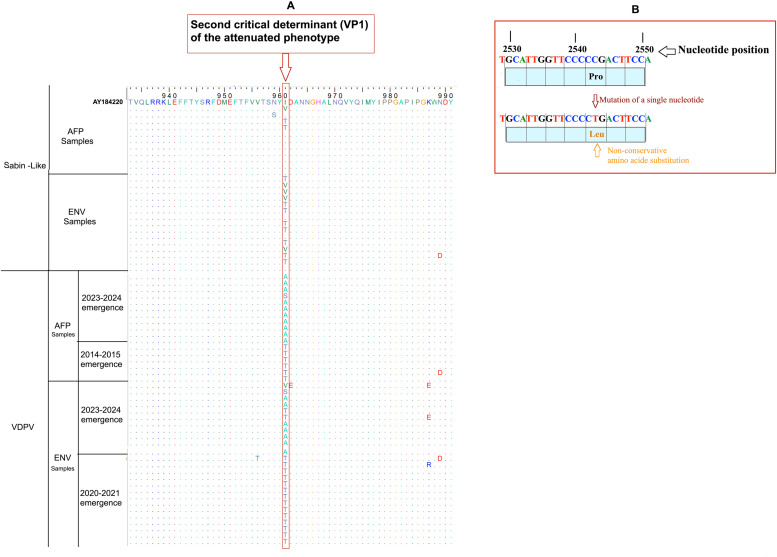
*In silico* analysis of amino acid (a) on the poliovirus type 2’s VP1 sequence. The conserved residues are indicated by a dot (.). The hotspot nucleotide mutation in the newly VDPV2 emergence group is identified by red box (b). AFP, acute flaccid paralysis; VDPV, vaccine-derived poliovirus type.

Accumulation of mutations play a key role in the evolution of PVs. *In silico* analysis of the nucleotide and amino acid of the PV type 2’s VP1 sequences, exhibited a polymorphism according to the cVDPV2 emergence group (Supplementary Material Figure S1). Currently, the second critical determinant of the attenuated phenotype on the VP1 gene (U2909C/A) corresponding to a substitution (Ile to Thr) was observed from all cVDPV2 isolates, including in three conservative substitutions (Ile to Val, Ile to Ala, and Ile to Ser) (Figure 2a). However, a single hotspot of nucleotide mutations (C2543T) was identified in the VP1 gene, resulting in an amino acid change from Proline to Leucine, particularly, on isolates that circulated between 2020-2021 and 2023-2024 (Figure 2b). These data suggest a possible impact of identified mutations on the mechanisms of emergence. Nevertheless, further studies assessing their impact on VDPV2’s virulence are warranted.

**Figure 3:**
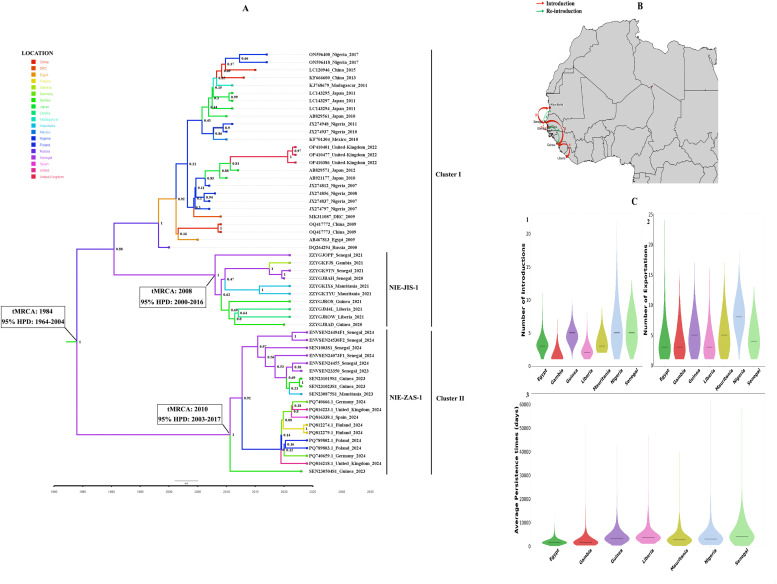
Bayesian inferred maximum clade credibility phylogenetic tree of complete VP1 (900 bp) of newly emerging cVDPV2 (a); map showing the spatiotemporal and geographical dispersal of the VDPV2 strains included in the present study (b); and estimated number of introductions (1), exportations (2), and average time of local persistence in days (3) for each African country (c). The Senegalese and Guinea cVDPV2 sequences obtained from the Intercountry Reference Center for Poliomyelitis at Institut Pasteur de Dakar were colored in purple and green, respectively. Tree branches are colored according to the country of origin and the estimated tMRCA and posterior probability (pp) are shown in nodes and the timescale in years is indicated in the x-axis. The horizontal black line denotes median estimates. cVDPV, circulating vaccine-derived poliovirus; tMRCA, time to the most recent common ancestor.

Furthermore, all recent cVDPV2s from Senegal belonged to the emergence group NIE-ZAS-1, as all 2024 European cVDPV2 isolates [4]. The emergence group NIE-ZAS-1 was first identified in Nigeria in July 2020 and has been described as circulating in many West African countries since 2021 [13]. Interestingly, the estimated tMRCA of cVDPV2 strains was around 1984 (95% Highest Posterior Density (HPD) range, 1964-2004) (Figure 3a). Interestingly, we identified two distinct cVDPV2 emergences in Senegal. Firstly, the 2020-2021 sequences from Senegal belonged to the NIE-JIS-1 emergence group which was introduced to Senegal around 2008 (95% HPD, 2000-2016) and formed a monophyletic group with sequences from the same period in the Republic of Guinea, Mauritania, Liberia, and The Gambia (cluster I). Secondly, the NIE-ZAS-1 emergence group emerged in Senegal around 2010 (95% HPD, 2003-2017) and was successively spread to the Republic of Guinea and Mauritania. However, the long stem branch of the NIE-ZAS-1 suggests an extended period of undetected circulation, potentially in an intermediate location, before its spread to Senegal. The 2023-2024 cVDPV2 sequences from Senegal formed a monophyletic subgroup with the 2023 sequences from the Republic of Guinea and Mauritania (cluster II) (Figure 3a). In addition, our data revealed that the NIE-ZAS-1 was recently introduced in Europe from Poland and originated from Senegal.

The spatio-temporal analysis showed that the cVDPV2 strains exhibited extensive dispersal in West Africa, especially between the Republic of Guinea, Mauritania, and Senegal, as well as between countries covering long distances (Figure 3a, 3b). Our data confirmed that the Republic of Guinea and Senegal seem to have been the key geographical sources for the recent cVDPV2 NIE-ZAS-1 emergence in the West African sub-region, with multiple cross-border transmissions, as previously reported during the 2020-2021 outbreak [5]. In addition, we found that Senegal exhibited the highest estimates regarding the number of introductions, exportations, and virus persistence compared with the other African countries (Figure 3c). In fact, the virus spread within Senegal and between Senegal and neighboring countries could be amplified by population movements, international travel, and cross-border trading activities. Considering the high risk for rapid geographical expansion of cVDPV2, it is then important for countries such as Senegal and the Republic of Guinea to strengthen surveillance to rapidly detect and curb the virus.

Therefore, it is essential to ensure vaccine accessibility, maintain uniformly high routine immunization coverage, and consider cross-country cooperation for simultaneous SIAs to minimize the consequences of poliovirus spread. Furthermore, a strengthened ES involving more sampling sites, especially in geographic regions with high risk of polio emergence, would support and facilitate poliovirus detection.

## Conclusion

The recent emergence of the VDPV2 NIE-ZAS-1 in Senegal points to the crucial need of an improved clinical surveillance strengthened with an expanded ES across the country for rapid control. In addition, ensuring a continuously high vaccine coverage relying on a combined OPV-IPV routine vaccination schema will help to mitigate the risk of future poliovirus re-emergence. The detection of cVDPV2 in sewage and AFP cases and their close genomic link with cVDPV2 strains from the Republic of Guinea underscores the importance of an improved cross-country cooperation for simultaneous vaccination campaigns and SIAs. An assessment of risk factors driving cVDPV2’s persistent circulation in Senegal is also warranted. In addition, there is a need for continuous genomic surveillance of polioviruses for rapid identification of emerging variants with large outbreak potential.

## Declaration of competing interest

The authors have no competing interests to declare.

## Appendix Supplementary materials

Image, application 1
