# Supplementary figures and images for: Recent emergence of vaccine-derived poliovirus type 2 in Senegal and virus spread to neighboring countries
Source: Int J Infect Dis. 2025 Dec;161:None. doi: 10.1016/j.ijid.2025.108122 (PMC12657269; doi:10.1016/j.ijid.2025.108122)

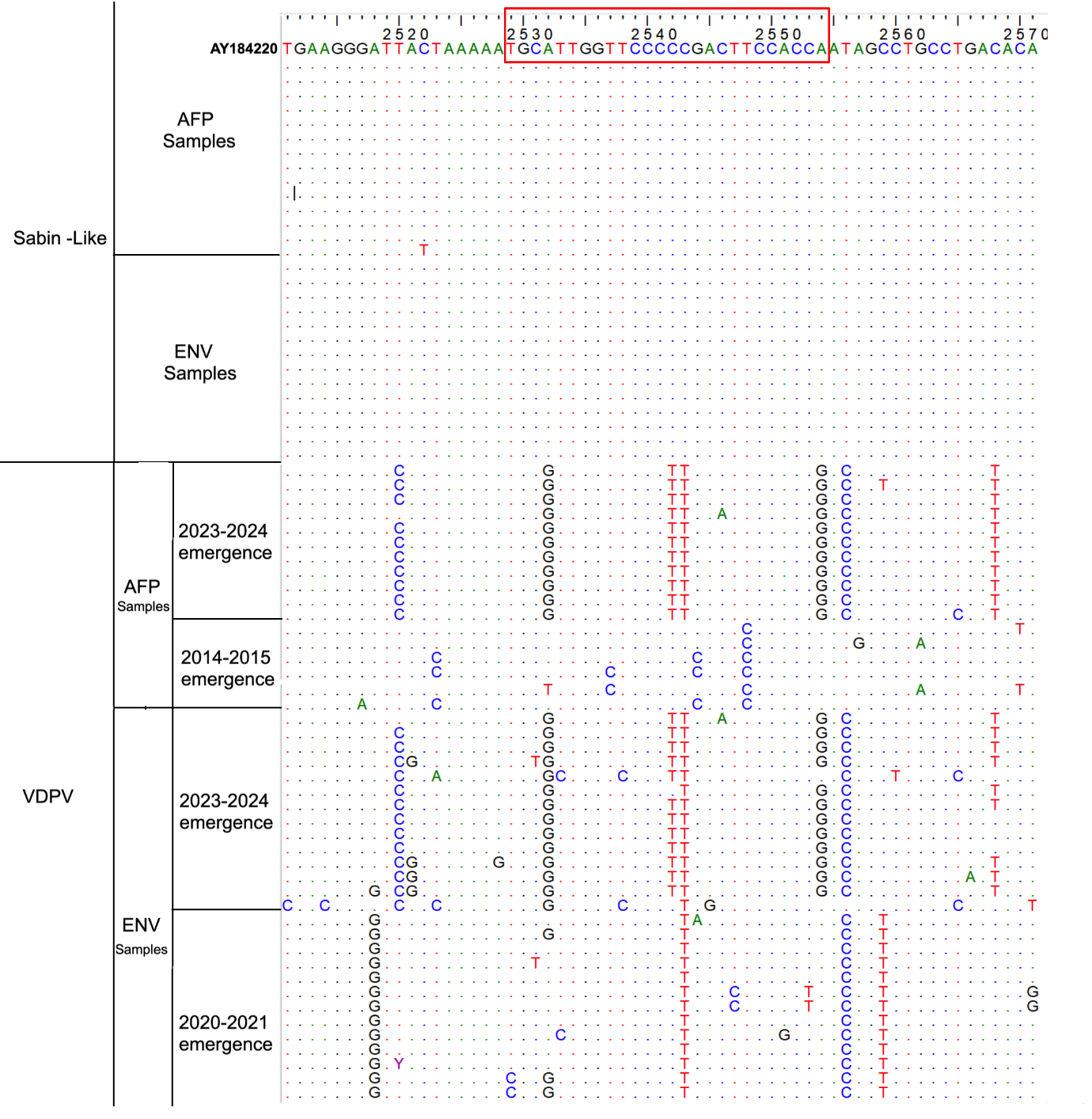

Supplement: Supplementary file 1 [file mmc1.docx]
